# Multiple generalist morphs of Lake Trout: Avoiding constraints on the evolution of intraspecific divergence?

**DOI:** 10.1002/ece3.2506

**Published:** 2016-10-05

**Authors:** Louise Chavarie, William J. Harford, Kimberly L. Howland, John Fitzsimons, Andrew M. Muir, Charles C. Krueger, William M. Tonn

**Affiliations:** ^1^ Center for Systems Integration and Sustainability Michigan State University East Lansing MI USA; ^2^ Department of Biological Sciences University of Alberta Edmonton AB Canada; ^3^ Cooperative Institute of Marine & Atmospheric Studies University of Miami Miami FL USA; ^4^ Fisheries and Oceans Canada Winnipeg MB Canada; ^5^ Fisheries and Oceans Canada Burlington ON Canada; ^6^ Great Lakes Fishery Commission Ann Arbor MI USA

**Keywords:** arctic, mixSIAR, niche partitioning, polymorphism

## Abstract

A generalist strategy, as an adaptation to environmental heterogeneity, is common in Arctic freshwater systems, often accompanied, however, by intraspecific divergence that promotes specialization in niche use. To better understand how resources may be partitioned in a northern system that supports intraspecific diversity of Lake Trout, trophic niches were compared among four shallow‐water morphotypes in Great Bear Lake (N65^°^ 56′ 39″, W120^°^ 50′ 59″). Bayesian mixing model analyses of stable isotopes of carbon and nitrogen were conducted on adult Lake Trout. Major niche overlap in resource use among four Lake Trout morphotypes was found within littoral and pelagic zones, which raises the question of how such polymorphism can be sustained among opportunistic generalist morphotypes. Covariances of our morphological datasets were tested against δ^13^C and δ^15^N values. Patterns among morphotypes were mainly observed for δ^15^N. This link between ecological and morphological differentiation suggested that selection pressure(s) operate at the trophic level (δ^15^N), independent of habitat, rather than along the habitat‐foraging opportunity axis (δ^13^C). The spatial and temporal variability of resources in Arctic lakes, such as Great Bear Lake, may have favored the presence of multiple generalists showing different degrees of omnivory along a weak benthic–pelagic gradient. Morphs 1–3 had more generalist feeding habits using both benthic and pelagic habitats than Morph 4, which was a top‐predator specialist in the pelagic habitat. Evidence for frequent cannibalism in Great Bear Lake was found across all four morphotypes and may also contribute to polymorphism. We suggest that the multiple generalist morphs described here from Great Bear Lake are a unique expression of diversity due to the presumed constraints on the evolution of generalists and contrast with the development of multiple specialists, the standard response to intraspecific divergence.

## 1 Introduction

Northern freshwater faunas have a number of interesting ecological and evolutionary characteristics, including substantial intraspecific diversification among and within individual lakes. This diversification has been facilitated by the low number of resident species that characterize these faunas, resulting in open niches and relaxed competition (MacDonald, Levy, Czarnecki, Low, & Richea, 2004; Robinson & Parsons, 2002; Skulason & Smith, 1995; Smith & Skulason, 1996). This intraspecific diversity represents “evolutionarily units” that greatly contribute to the freshwater biodiversity of these northern species depauperate regions.

Northern freshwater fishes, in particular, have been informative for studying divergence due to numerous examples of intraspecific diversification across a range of coexisting taxa (Mcphee, Noakes, & Allendorf, 2012). The process of divergence represents a continuum of outcomes. At one extreme are found unstable systems with flexible and highly plastic populations with gene flow. At the other extreme are found genetically distinct morphs, which through adaptation to niche use and reproductive isolation seem to have lost some of the original capacity for plasticity (Nosil, 2012; Oke et al., 2016; Snorrason & Skúlason, 2004). Mechanisms of flexibility are expected to be evolutionary costly and that, as ecosystems stabilize and become more predictable, generality and plasticity should be lost, whereas specialization and genetic divergence should increase (Bolnick et al., 2003; Snorrason & Skúlason, 2004; Svanbäck, Quevedo, Olsson, & Eklöv, 2015; Van Kleunen & Fischer, 2005).

Intraspecific polymorphisms within lake and stream systems are considered unique because of adaptive shifts in resource use within a species that are found in one locality and then repeated consistently across many systems (i.e., parallel evolution) (Klemetsen, 2013). Most cases of polymorphism in freshwater fishes are linked to discrete habitats and foraging opportunities, such as littoral and pelagic niches (Faulks, Svanbäck, Eklöv, & Östman, 2015; Parker, Stepien, Sepulveda‐Villet, Ruehl, & Uzarski, 2009; Præbel et al., 2013). Along with segregation by habitat and diet, important intraspecific differences among morphotypes in life history, genetics, and behavior have also been observed (Hansen et al., 2016; Schluter & McPhail, 1993; Skulason & Smith, 1995). Patterns of ecological specializations within fish species have been frequently reported in the past decade (Klemetsen, 2010; Muir, Hansen, Bronte, & Krueger, 2015; Robinson & Parsons, 2002). Some of these identify novel forms of resource polymorphism, such as a profundal Lake Whitefish (*Coregonus lavaretus*), in addition to the more typical divergence across littoral–pelagic habitats (Præbel et al., 2013).

Lake Trout (*Salvelinus namaycush*) in Great Bear Lake is an example of novel polymorphism, lacking the depth partitioning commonly associated with Lake Trout differentiation elsewhere (Eshenroder, 2008; Zimmerman, 2006; Zimmerman, Krueger, & Eshenroder, 2007; Zimmerman, Schmidt, Krueger, Vander Zanden, & Eshenroder, 2009). Indeed, considerable intraspecific diversity and plasticity have been documented within the shallow‐water regions of Great Bear Lake (Alfonso 2004; Blackie et al. 2003;Chavarie, Howland, Harris, & Tonn, 2015; Chavarie, Howland, & Tonn, 2013; Chavarie, Howland, Venturelli et al., 2016). Such shallow‐water diversity could be an undervalued characteristic of the species across its broader geographic range, for example, historical anecdotes of similar polymorphisms only exist for the Laurentian Great Lakes (Brown, Eck, Foster, Horrall, & Coberly, 1981; Goodier, 1981). The shallow‐water polymorphisms in Great Bear Lake may be maintained by multiple levels of habitat partitioning and differences in resource use (Chavarie, Howland, Gallagher, & Tonn, 2016), typically involving two axes (i.e., littoral–pelagic or littoral–profundal) of adaptive divergence associated with variable resources (Præbel et al., 2013).

**Figure 1:**
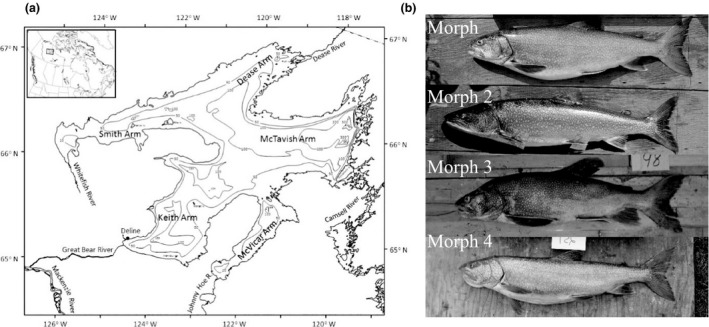
(a) Map of Great Bear Lake, Northwest Territories, Canada, adapted from Johnson (1975) and Chavarie, Howland, Harris et al. (2015), indicating general bathymetry and its major rivers. Insert: location of study area within Canada. (b) The four shallow‐water morphotypes of Lake Trout from Great Bear Lake identified in Chavarie et al. (2013), Chavarie, Howland, Harris et al. (2015); Chavarie, Howland, Venturelli et al. (2016), Chavarie et al. (2016) : the generalist, the piscivore, the benthic‐oriented, and the pelagic specialist, morphs 1–4, respectively.

Within the shallow‐water (≤30 m) zones of Great Bear Lake, four morphs differed in head, body, and fin morphology (Figure 1) (Chavarie, Howland, Harris et al., 2015; Chavarie, Howland, Venturelli et al., 2016; Chavarie et al., 2013). Morph 1 was characterized by a smaller head and intermediate fins, Morph 2 had the largest head and jaws but smallest fins, Morph 3 had the longest fins and a robust body shape, and Morph 4 had a thick curved lower jaw and the smallest caudal peduncle of the morphs. Based on analyses of fatty acids and stomach contents, Morph 1 was defined as a generalist, Morph 2 had higher proportions of fish in its diet than the other morphs, Morph 3 was a benthic‐oriented generalist, and Morph 4 was regarded as a pelagic specialist (Chavarie et al., 2016). Adult growth rates, age and size at maturity, and survival rates differed among morphs, consistent with predictions from foraging theory. Reduced somatic growth and higher reproductive investment were found in the generalist morph (Morph 1), high growth rates throughout life characterized the piscivorous morph (Morph 2), and intermediate life histories defined the more benthic‐ and pelagic‐oriented morphs (Morph 3 and Morph 4) (Chavarie, Howland, Venturelli et al., 2016). Finally, the four morphotypes showed some genetic differentiation from one another, especially Morph 2 when compared to the three other morphs (Harris et al., 2015). However, morphotypes were genetically more similar to one another when compared with populations from outside Great Bear Lake, supporting an intralake model of divergence (Harris et al., 2015) (Table 1).

**Table 1:** Synthesis of studies conducted on Lake Trout in Great Bear Lake (Chavarie, Howland, Harris et al. (2015); Chavarie, Howland, Venturelli et al. (2016); Chavarie et al., 2013; Chavarie et al. 2016; Harris et al., 2015)

	Genetics	Fatty acids	Stomach contents	Life history
Morph 1	*Pairwise F*_ST_: Morph 2:0.0063Morph 3:0.0038Morph 4:0.012	No pattern, variable signatures	Schoener's: all >0.60*E*:0.71	*Age median*: 20.0 ± 0.45*Age at maturity*: 17.4 ± 2.3*Length median*: 641.7 ± 4.97
Morph 2	*Pairwise F*_ST_:Morph 3:0.0067Morph 4:0.017	Divided into two groups: Pelagic (14:0, 18:2n‐6, 18:3n‐3, 18:4n3, 18:1n‐9, 20:1n‐7, and 22:1n‐9) and benthic–cannibalistic (16:0, 18:0, 20:4n‐6, and 22:6n‐3)	*Schoener's*: Morph 3 = 0.73; Morph 4 = 0.39*E*:0.68	*Age median*: 22.0 ± 0.39*Age at maturity*: 20.2 ± 7.3*Length median*: 670.8 ± 5.16
Morph 3	*Pairwise F*_ST_:Morph 4:0.0086	Variable signatures but a weak benthic division (16:1n‐7, 16:2n4, 16:3n‐4, 18:1n7, 18:2n4, 18:3n6, and 22:1n7)	*Schoener's*: Morph 4 = 0.56*E*:0.69	*Age median*: 29.0 ± 0.86*Age at maturity*: 18.6 ± 1.9*Length median*: 644.0 ± 5.12
Morph 4		Most specialized (clustered signatures) and pelagic (14:0, 18:2n‐6, 18:3n‐3, 18:4n3, 18:1n‐9, 20:1n‐7, and 22:1n‐9)	*Schoener's*: all ≤0.61*E*:0.99	*Age median*: 26.0 ± 1.51*Age at maturity*: 20.2 ± 0.4*Length median*: 683.0 ± 11.89

“Genetics” (*n* = 473) are pairwise *F*_ST_ values based on 24 microsatellite loci. “Fatty acids” (*n* = 41) provide details on the major dietary fatty acids driving PCA discrimination among morphs (*n* = 126) and observed patterns. “Stomach contents” (*n* =92) provide Schoener's overlap index (values higher than 0.6 represent biologically significant diet overlap) and mean pairwise diet dissimilarity between individuals (E), ranging from zero (absence of interindividual niche difference) to one (complete interindividual variation). Only two stomachs were analyzed for Morph 4. “Life history” (*n* = 902) includes age (years) and length (mm) medians among morphs and an estimated age at maturity (years) from biphasic models.

Overlap and seasonality of diets, and similarity in habitat use among the four morphs led us to question the degree to which trophic and habitat partitioning has driven this divergence (Chavarie et al., 2016). Although these Lake Trout are at a young stage of differentiation (i.e., <2,000 years; Harris et al., 2015), the lack of major differences in habitat and diet was unexpected because sympatric differentiation is typically linked to easily identifiable resource‐based segregation (Skúlason, Snorrason, & Jonsson, 1999; West‐Eberhard, 2003, 2005).

Stable carbon (δ^13^C) and nitrogen (δ^15^N) isotopes are ecological tracers that can complement stomach content and fatty acids data (Beaudoin, Tonn, Prepas, & Wassenaar, 1999; Boecklen, Yarnes, Cook, & James, 2011; Layman et al., 2012). Although using two dietary methods that reflect longer time frames than the snapshot of stomach contents may appear redundant, the different perspectives brought by fatty acids and stable isotope analyses in describing resource use of opportunistic feeders, such as Lake Trout, ensure a more comprehensive description of trophic habits. Stable isotopic ratios represent broad patterns in resource use and food web structure, with distinct δ^13^C values associated with different sources of primary production (e.g., pelagic vs. littoral) and δ^15^N associated with different trophic levels (3–4‰ enrichment per trophic level) (Post, 2002). Consistent with Hutchinson's (1957) notion that an ecological niche can be represented by a multidimensional hypervolume, stable isotopes have been used recently to delineate trophic niche axes and to quantify niche space (Bolnick et al., 2003; Jackson, Inger, Parnell, & Bearhop, 2011; Newsome, Martinez Del Rio, Bearhop, & Phillips, 2007). Stable isotopic ratios of organisms track energy sources (Hobson, 1999) and provide important information about energy flux through food webs (Post, 2002). Consequently, isotopic mixing models can be used to estimate proportions of prey from different sources contributing to resource use (Eloranta, Siwertsson, Knudsen, & Amundsen, 2011; Pomerleau et al., 2011).

Given the uncertainty regarding the roles of diet and habitat as drivers of divergence for the shallow‐water Lake Trout of Great Bear Lake, the goal of this study was to compare resource use among the four morphotypes using an isotopic mixing model. Specifically, we (1) evaluated resource use among morphotypes to determine whether their habitat partitioning and foraging opportunities were consistent with the theory of resource polymorphism. Subsequently, we (2) compared the level of trophic specialization of each morph and their overlap to each other using isotopic niche areas. Finally, we (3) explored relationships between morphology and trophic adaptations (i.e., form–function relationships) among morphs to investigate whether patterns between stable isotopic values (δ^13^C and δ^15^N) were directly related to differences in morphology. Determining whether Lake Trout morphotypes from Great Bear Lake represent distinct trophic units should advance our understanding of the role of resource segregation in the development and maintenance of sympatric divergence.

## 2 Materials and Methods

### 2.1 Study system and data collection

Great Bear Lake is the world's ninth largest and 19th deepest lake, with a complex, multi‐armed surface area of 31,790 km^2^ and a maximum depth of 446 m (mean depth = 90 m). The lake is located in Canada's Northwest Territories, on the Arctic Circle, between 65 and 67°N latitude and 118 and 123°W longitude. The lake's limnological and biological characteristics are typical of Arctic freshwater systems, with low productivity and interspecific diversity (Johnson, 1975), but considerable intraspecific diversity within Lake Trout (Chavarie et al., 2013; Chavarie, Howland, Harris et al., 2015; Chavarie, Howland, Venturelli et al., 2016; Chavarie et al., 2016) and Cisco (*Coregonus artedi*) (Howland et al., 2013).

**Figure A1:**
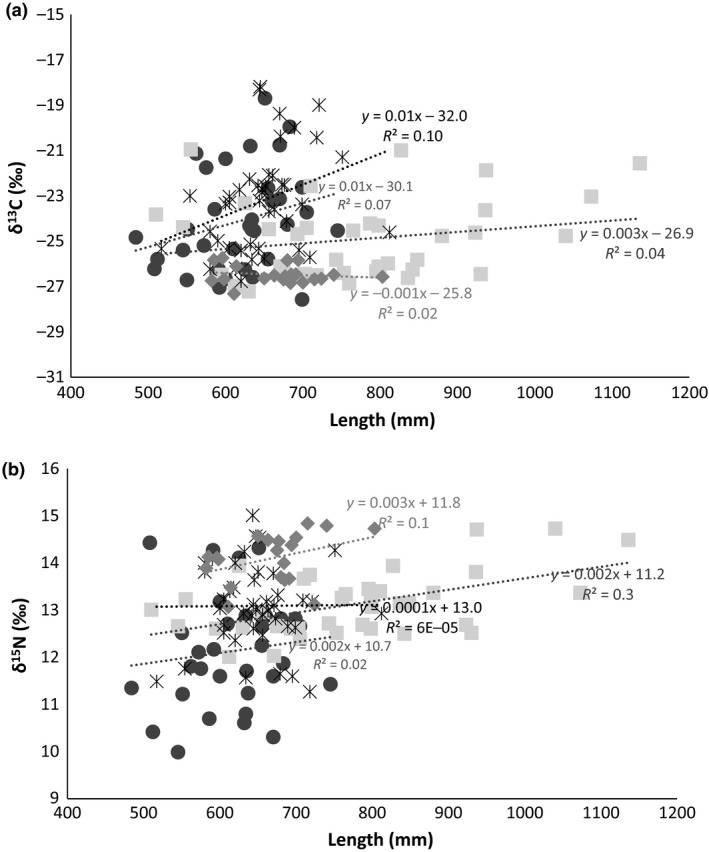
Regression plots of δ^13^C (a) and δ^15^N (b) against length of Lake Trout sampled in the shallow‐water zone (≤30 m) of Great Bear Lake. For δ^13^C, Morph 3 had a regression that differed significantly from 0 (*p* ≤ .05) with a *R* ^2^ = .1, whereas regressions for the other morphs were not significant (*p* ≥ .05). When all morphs were combined, the overall regression also did not differ from 0 (*p* ≥ .05). For δ^15^N, Morph 2 had a regression that differed significantly from 0 (*p* ≤ .05) with a *R* ^2^ = .3, whereas regressions for the other morphs were not significant (*p* ≥ .05). When all morphs were combined, the overall regression differed from 0 (*p* ≤ .05) with a *R* ^2^ = .07

**Figure A2:**
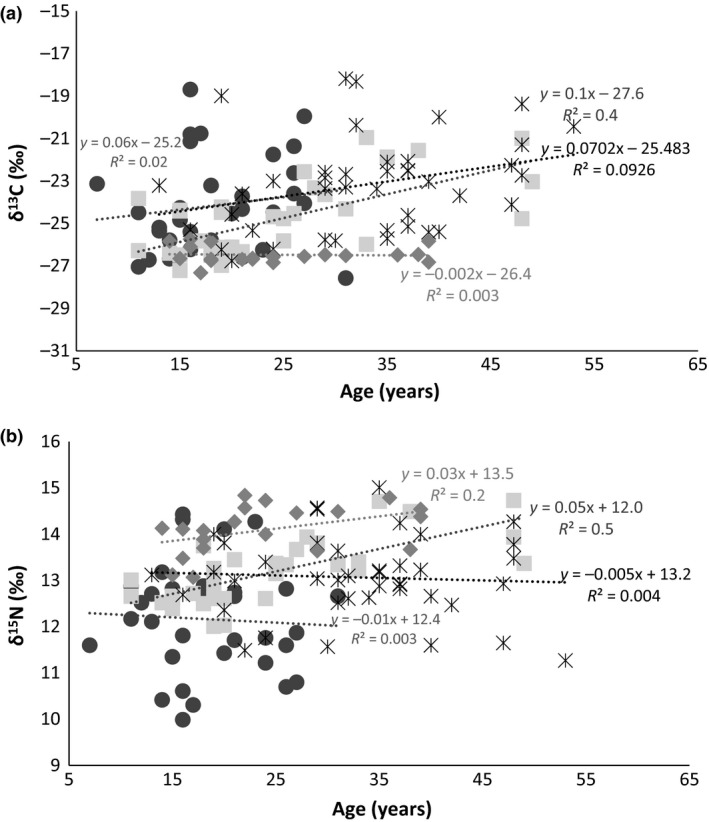
Regression plots of δ^13^C (a) and δ^15^N (b) against age of Lake Trout sampled in the shallow‐water zone (≤30 m) of Great Bear Lake. For δ^13^C, Morph 2 had a regression that differed significantly from 0 (*p* ≤ .05) with a *R* ^2^ = .4, whereas other morphs had no significant regressions (*p* ≥ .05). When all morphs were combined, the overall regression differed from 0 (*p* ≥ .05) with a *R* ^2^ = .1. For δ^15^N, Morph 2 had a regression that differed significantly from 0 (*p* ≤ .05) with a *R* ^2^ = .5, whereas regressions for other morphs were not significant (*p* ≥ .05). When all morphs were combined, the overall regression differed from 0 (*p* ≤ .05) with a *R* ^2^ = .06

Gill netting was conducted in all five arms of the lake during July and August of multiple years at depths ≤30 m (see Chavarie et al., 2016 for details). Morphs were assigned using a lateral full‐body digital image and a multivariate assignment method based on morphology (see Chavarie et al., 2013; Chavarie, Howland, Harris et al., 2015; Chavarie, Howland, Venturelli et al., 2016). Other variables recorded were fork length (mm), weight (g), sex, and stage of maturity (juvenile, mature, and resting categories; Chavarie et al., 2013). A dorsal muscle sample was removed and frozen at −20°C for stable isotope analysis. We focused on adult trout due to the difficulty of classifying juveniles into morphs (Chavarie et al., 2013) and to avoid the confounding effects of ontogenic shifts in morphology and diet. Thus, juvenile Lake Trout were considered only as potential prey of adults (Chavarie et al., 2016). For this study, stable carbon and nitrogen isotopes were analyzed on muscle tissue from 133 Lake Trout (Morph 1 = 34, Morph 2 = 37, Morph 3 = 42, and Morph 4 = 20; morphs classified in Chavarie, Howland, Harris et al., 2015; Chavarie, Howland, Venturelli et al., 2016; Chavarie et al., 2013), of which 126 were previously analyzed for fatty acids (Chavarie et al., 2016). Lake Trout from Great Bear Lake do not display sexual differences in morphology and life history (see Chavarie, Howland, Venturelli et al., 2016; Chavarie et al., 2013); thus, sexes were pooled. Length and age characteristics were selected to be as similar as possible among morphs. Morph 2 was longer and Morph 3 was older than the other morphs, which is consistent with their life histories (Chavarie, Howland, Venturelli et al., 2016; Table 1). δ^13^C and δ^15^N values were plotted against length and age to test for their effects on isotope values (see Figures A1 and A2 in Appendix). No major patterns were detected among or within morphs, except for Morph 2, where isotope values had some degree of linear relationship with age (Figure A2 in Appendix). Consequently, size and age classes of morphs were pooled.

Cisco, Lake Whitefish, Round Whitefish (*Prosopium cylindraceum*), and juvenile Lake Trout were collected as bycatch in gill nets used to capture adult Lake Trout. Arctic Grayling (*Thymallus arcticus*) were caught by angling, and stickleback were caught with minnow traps and by seining. Horizontal (5 min) and vertical (10 hauls) tow nets (50 cm diameter, 100 μm mesh net) from littoral (~5 m depth) to pelagic zones (≤30 m) collected zooplankton. A sweep net (in depths up to 1 m; 500 μm) and petite ponar grabs (≤30 m) collected macro‐invertebrates. All invertebrates were held for 24 hr to allow gut evacuation; only the soft body tissue of molluscs was prepared for stable isotope analysis. Finally, items (e.g., terrestrial invertebrates, *Mysis*, sculpins) collected from Lake Trout stomach contents were washed and used for isotopic analyses to complete prey collection. Prey identification ranged from species to family (Table 2).

**Table 2:** Prey groupings used in mixSIAR analyses (see text). “Source taxa” represents the taxa with measured isotopic values that were included in the prey model. Chironomidae was separated from other Diptera due to its importance as prey (Hulsman et al., 2016). Inv. is invertebrate

Prey groups	Source taxa
Littoral inv.	*Diporeia;* Gammaridae*; Hyalella*;* Monoporeia*
Littoral inv.	Chironomidae
Littoral inv.	*Callibaetis*;* Heptagenia*
Littoral inv.	*Cypriconcha*
Littoral inv.	*Kogotus*;* Nemoura*
Littoral inv.	*Agrypnia*;* Asynarchus*;* Limnophilus*;* Phrygarea*; unknown Trichoptera *Psychoglypha*
Littoral inv.	*Oreodytes*;* Stictotarsus*
Littoral inv.	*Leptotarsus*
Littoral inv.	Corixidae
Littoral inv.	Terrestrial inv.: Empididae; *Tabanus;* Formicidae; Lepidoptera; Orthoptera
Littoral shelled inv.	*Gyraulus*;* Lymnaea*;* Physa*
Littoral shelled inv.	*Pisidium*;* Valvata*
Littoral fish	Arctic Grayling
Littoral fish	Lake Whitefish
Littoral fish	Stickleback
Littoral fish	Round Whitefish
Littoral fish	Slimy Sculpin
*Mysis*	*Mysis*
Cisco	Cisco
Juvenile Lake Trout	Juvenile Lake Trout

### 2.2 Stable isotopes

Lake Trout muscle and prey samples were freeze‐dried for 24 hr and homogenized into a fine powder. Dried samples were weighed (1.0 ± 0.1 mg) into tin capsules and shipped to the University of Saskatchewan, Department of Soil Sciences, for stable carbon and nitrogen isotope analysis. Carbon and nitrogen stable isotopes were measured with an ANCA G/S/L elemental analyzer coupled to a Tracer/20 mass spectrometer (Europa Scientific, Crewe, UK). The standard error from the mean of each isotopic run never exceeded 0.05 ‰.

Stable isotope results were expressed in delta (δ) notation (as parts per mil (‰)), the normalized ratio of a sample to an internationally accepted standard. The standards were Vienna Pee Dee Belemnite for δ^13^C and atmospheric nitrogen for δ^15^N. An internal reference was egg albumen, and the *SD* of reference material was 0.05‰ for δ^13^C and 0.08‰ for δ^15^N. Due to high C:N ratios (>3.5), indicating high‐lipid content, the fish δ13C values were lipid‐corrected following Post et al. (2007).

### 2.3 Statistical analysis

Analyses of variance (ANOVA) followed by Bonferroni post hoc correction were performed with Systat v. 13 (Systat Software Inc., Chicago, IL, USA) on muscle δ^13^C and δ^15^N values to determine whether the four morphotypes differed in their isotopic values.

**Figure A3:**
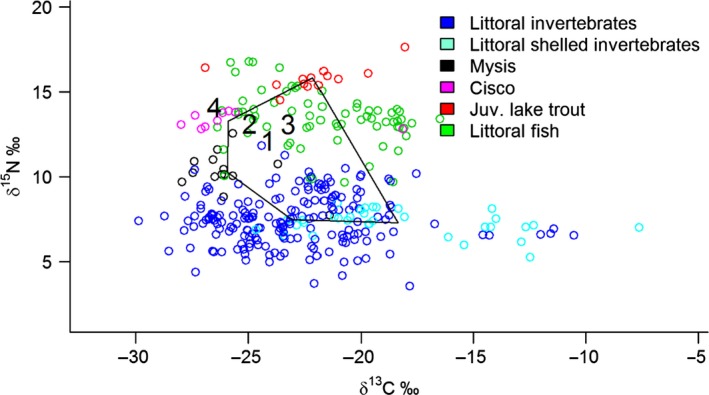
Mixing models source geometry of isotopic values of consumer morphs (1–4) and the distribution of prey isotopic values. The vertices of the convex hull are the means of each prey group for prey

Prey contributions to Lake Trout diet were estimated using isotopic mixing models (Moore and Semmens 2008, Parnell, Inger, Bearhop, & Jackson, 2010). Prey taxa were combined into coarse diet groups according to three criteria: major prey items previously identified in stomach analyses, environmental gradients reflecting shallow‐water habitat use (i.e., a littoral–pelagic axis), and by major taxonomic category (i.e., fish or invertebrates) (Bjorkland et al., 2015; Chavarie et al., 2016; Francis et al., 2011). Combining prey items accordingly produced a prey model consisting of littoral fish, littoral invertebrates, littoral shelled invertebrates, *Mysis*, juvenile Lake Trout, and Cisco (Table 2). A reduction of prey taxa into these aggregate groupings was required to ensure more precise estimates of dietary fractions that could be reasonably estimated using isotope mixing models (Phillips et al., 2014). A Bayesian mixing model estimated proportional contributions of prey groups to diets of Lake Trout morphs 1–3. Morph 4 was excluded from mixing models because source geometry did not encompass this morph within the distribution of prey isotopic values (Figure A3 in Appendix). Several alternative prey model formulations were initially considered; however, none could produce prey isotopic value distributions that would encompass Morph 4 isotopic values.

Mixing model analyses were conducted in the R (R Core Team 2012) environment using the mixSIAR library (Stock & Semmens, 2013). Trophic enrichment was adjusted using trophic enrichment factors (TEF) of 0.39 ‰ (*SD* 1.69) for δ^13^C and 3.4 ‰ (*SD* 0.9604) for δ^15^N (Post, 2002). Development and implementation details of Markov chain Monte Carlo (MCMC) fitting algorithms used in mixSIAR are described by Parnell (2010, 2013), Stock and Semmens (2013). In brief, the Bayesian framework generates estimates of predator dietary proportions from each prey source group as a posterior probability distribution (Parnell et al., 2010). Mixing models for each morphotype were specified to use diffuse priors on dietary fractions (i.e., a Dirichlet distribution with *alpha *= *c*(1,1,…1), as specified in the mixSIAR user interface) and to include a residual error term, but did not include process error or parametrization for individual‐level variation (Parnell et al., 2013). After discarding an initial 200,000 iterations, the Markov chain Monte Carlo (MCMC) algorithm converged for all models, as Gelman–Rubin criteria for each parameter were <1.05 (Gelman, Carlin, Stern, & Rubin, 2014). Approximation of the posterior distribution was obtained from a subsequent 100,000 iterations from three parallel chains and a thinning rate of every 100th sample.

Niche region dimensions and pairwise niche overlap of morphotypes were obtained using the probabilistic method developed by Swanson et al. (2015), which is available in the nicheROVER R library. Their approach estimates parameters of the multivariate normal distribution, allowing isotopic niche dimensions to be defined as probability regions in multivariate space. Uncertainty in niche regions is accounted for using a Bayesian inference framework (Swanson et al., 2015). Ellipses representing 95% probability niche regions were generated using the posterior expectation of the bivariate normal distribution estimated using the Bayesian approach in nicheROVER. Percentage niche overlap was calculated in nicheROVER using respective 95% niche regions between each pair of morphs. Niche overlap is defined as the probability that an individual from one morph is found within the niche region of a second morph (Swanson et al., 2015). Uncertainty in niche overlap was reported as the posterior distribution of overlap percentage along with the Bayesian 95% credible intervals for each pairwise morph comparison.

Morphological data for the 133 Lake Trout were quantified from photographed fish based on twenty‐three landmarks, twenty semilandmarks, and twelve linear distances from Chavarie, Howland, Harris et al. (2015), Chavarie, Howland, Venturelli et al. (2016), Chavarie et al. (2013) so as to extract body and head shape, and linear measurements. All morphological measurements were independent of size, using centroid sizes or residuals from regression on standard length (Zelditch, Swiderski, Sheets, & Fink, 2004). The first principal component (PC) scores from principal component analyses (PCA) of morphological data (body and head shape using PCAGEN; IMP software, and linear measurements using PC‐ORD; McCune & Mefford, 2011; Zelditch et al., 2004) were plotted against δ^13^C and δ^15^N values to infer the relationships between variation in morphology and trophic adaptations (i.e., form–function relationships) (Bock & Von Wahlert, 1965; Cooke & Terhune, 2015; Lauder, 1981). To examine whether morphological variation among morphotypes was influenced by habitat partitioning and/or trophic position, δ^13^C values were selected to distinguish between littoral versus pelagic reliance (i.e., vertical or horizontal habitat partitioning) (Post, 2002) and δ^15^N values were used to discriminate trophic positions in the food web (i.e., omnivory to piscivory) (see Paull, Martin, & Pfennig, 2012). A two‐block partial least‐squares analysis with 10,000 permutations using PLSMaker8 from IMP programs (http://www3.canisius.edu/~sheets/morphsoft.html) (Zelditch et al., 2004) was conducted on δ^13^C and δ^15^N to test the covariance of body and head shape and ecological variables. Slopes of linear regressions were tested for differences from 0 for traditional linear measurements.

## 3 Results

**Figure 2:**
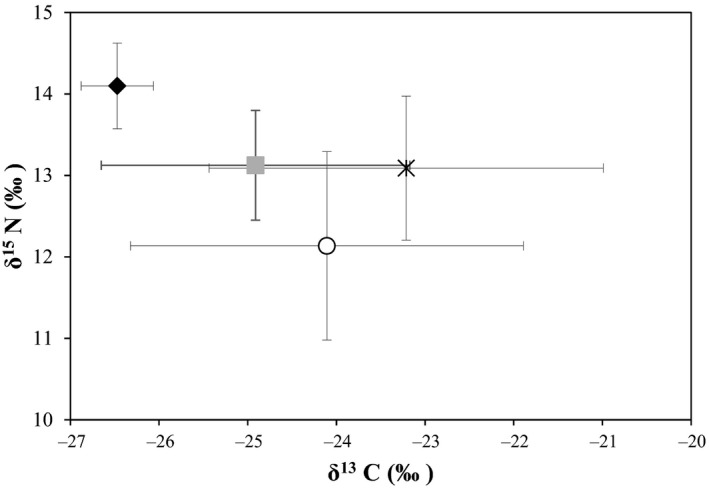
Biplot for muscle mean δ^15^N ‰ versus δ^13^C ‰ ± SD for four morphotypes of Lake Trout from Great Bear Lake, represented as open circle = Morph 1, light gray square = Morph 2, X = Morph 3, and black diamond = Morph 4.

Mean (± SD) muscle isotope values of Lake Trout morphotypes ranged from −26.5 ‰ ± 0.4 (Morph 4) to −23.2 ‰ ± 2.2 (Morph 3) for δ^13^C and from 12.1 ‰ ± 1.2 (Morph 1) to 14.1 ‰ ± 0.5 (Morph 4) for δ^15^N (Figure 2; Table A2 in Appendix). Muscle δ^13^C values differed among morphotypes (ANOVA, *df* = 3, *p* ≤ .05) with Morph 4 (more pelagic) differing significantly from all other morphs; Morph 3 (more benthic) also differed from Morph 2 (*p* ≤ .05). All morphs differed from each other for δ^15^N except Morph 2 and Morph 3 (*p* ≤ .05); Morph 4 was at the highest trophic level and Morph 1 at the lowest.

**Figure 3:**
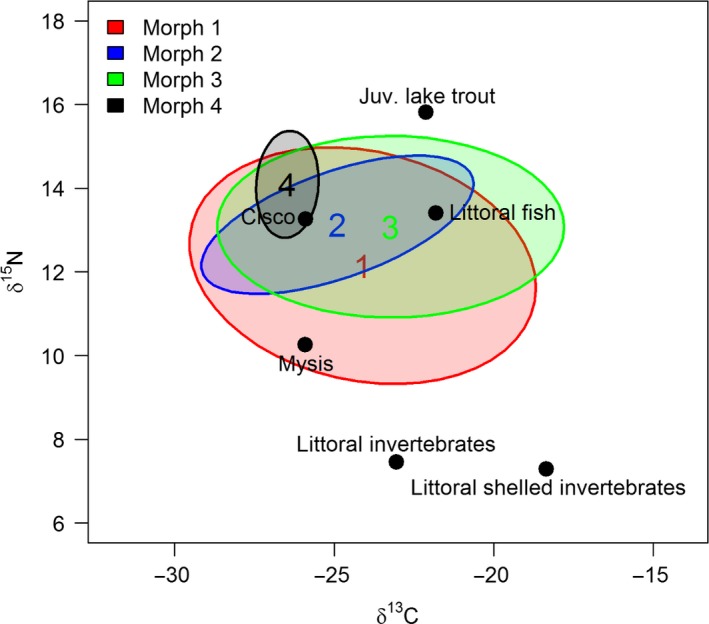
Probabilistic (95%) niche regions of carbon (δ^13^C ‰) and nitrogen (δ^15^N ‰) for four shallow‐water morphs of Lake Trout in Great Bear Lake. Potential prey is shown as black filled circles, and each Lake Trout morph is represented by a shaped ellipse.

**Figure 4:**
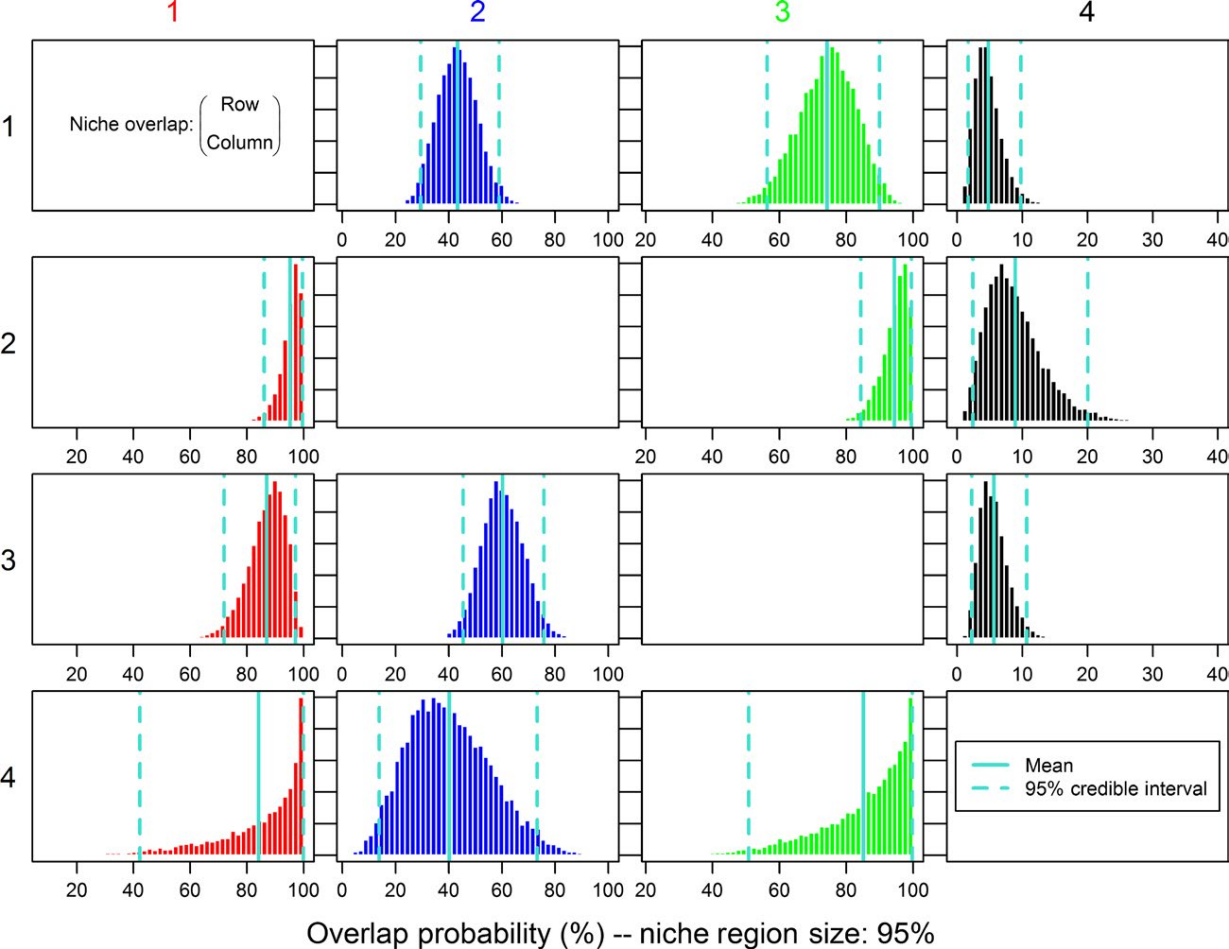
Posterior distribution of percentage overlap of 95% niche regions among Lake Trout morphotypes. Niche overlap is defined as the probability that an individual from one morph (rows) is found within the niche region of a second morph (columns).

The range of stable isotope values of potential prey sources was wide but within the range of isotope values observed among the shallow‐water morphotypes (Figure 3; Figure A3 in Appendix). Potential prey spanned over 20 ‰ for δ^13^C and over 10 ‰ for δ^15^N (Figure 3; Table A1 in Appendix). The main prey of Morph 1 was Cisco and *Mysis*, although juvenile Lake Trout and littoral fish were also relatively important (Table 3). Juvenile Lake Trout and especially Cisco dominated the resource use of Morph 2, while Lake Trout juveniles, littoral fish, and Cisco were most important for Morph 3 (Table 3). Contributions of other prey were smaller (<0.15) but varied among morphs. The isospace plot from mixSIAR suggested considerable overlap among the shallow‐water morphotypes, with some differences in niche widths and/or positions (Figure 3). NicheROVER calculated smaller niches for Morph 2 and Morph 4 than for the other morphs. Individuals from Morph 1 and Morph 3 had low–moderate probabilities of sharing the same niche spaces as Morph 2 and Morph 4 (Figure 4, Table 4). Conversely, there are intermediate–high probabilities of finding individuals of Morphs 2 and 4 within the niche spaces encompassed by Morphs 1 and 3 (Figure 4, Table 4).

**Table 3:** Mean (*SD*) diet fractions of prey for each Lake Trout morph 1–3, from mixSIAR models

Prey groups	Predator morph		
	Morph 1	Morph 2	Morph 3
Littoral fish	0.15 (0.11)	0.10 (0.08)	0.21 (0.16)
Littoral invertebrates	0.10 (0.07)	0.04 (0.03)	0.07 (0.05)
Littoral shelledinvertebrates	0.06 (0.05)	0.03 (0.02)	0.05 (0.04)
*Mysis*	0.21 (0.13)	0.10 (0.08)	0.12 (0.09)
Juvenile Lake Trout	0.16 (0.10)	0.20 (0.10)	0.33 (0.13)
Cisco	0.31 (0.17)	0.54 (0.14)	0.21 (0.13)

**Table 4:** Probabilistic niche overlap values (mean overlap ± *SD*) calculated from nicheROVER using 95% niche regions between each pair of Lake Trout morphs from Great Bear Lake (Swanson et al., 2015)

	Morph 1	Morph 2	Morph 3	Morph 4
Morph 1		0.43 ± 0.07	0.75 ± 0.09	0.05 ± 0.02
Morph 2	0.95 ± 0.04		0.95 ± 0.04	0.09 ± 0.05
Morph 3	0.87 ± 0.07	0.60 ± 0.08		0.06 ± 0.02
Morph 4	0.84 ± 0.15	0.40 ± 0.15	0.84 ± 0.15	

Rows represent the probabilities (≤1) of finding an individual of each Morph within the niche region of another morph.

**Figure 5:**
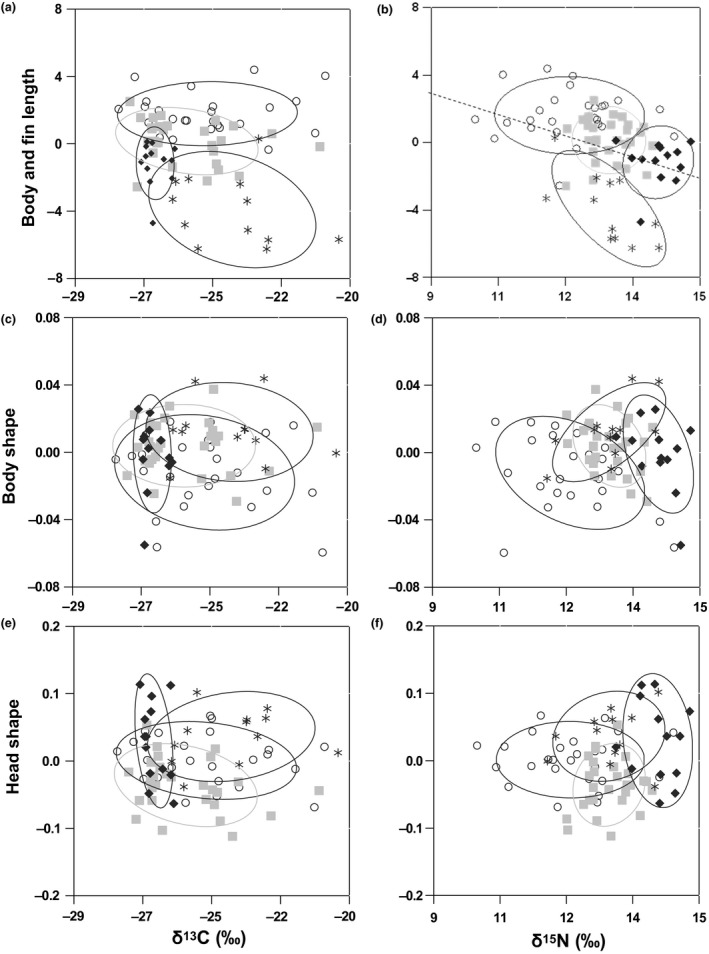
Scores of the first principal component for fin and body lengths (a, b; representing 37% of the variation), body shape (c, d; 39%), and head shape (e, f; 45%) of four shallow‐water Lake Trout morphotypes from Great Bear Lake (Chavarie, Howland, Harris et al. (2015)), plotted against carbon (δ^13^C ‰) and nitrogen (δ^15^N ‰) stable isotope values. Morphs were identified by McClust cluster analysis (Fraley & Raftery, 2009). The four shallow‐water morphotypes of Lake Trout from Great Bear Lake are represented as follows: open circle = Morph 1, light gray square = Morph 2, X = Morph 3, and black diamond = Morph 4. Each morph is also outlined by a 68.3% confidence ellipse.

Form–function analyses (i.e., morphology‐trophic adaptation patterns) suggested that morphological variations were more strongly associated with trophic level (δ^15^N) than with a littoral–pelagic reliance (δ^13^C). Morphological PCs plotted against δ^13^C values did not show significant relationships among the morphs 1–3, whereas Morph 4 had lower (more pelagic) and less variable δ^13^C values than the other three morphs (Figure 5a,c,e). Similarly, although the two‐block partial least‐squares analyses found significant relationships for littoral–pelagic reliance (δ^13^C) versus body and head (body: *r* = .32; permutation test, *p* ≤ .01; head: *r* = .27; permutation test *p* ≤ .01), the first singular axis (SA) did not differ from that expected by chance (body: eigenvalues coefficient = .0046; permutation test, *p* = .77; head: eigenvalues coefficient = .020; permutation test, *p* = .32); this occurs when the axis explains a trivial part of the covariance. In contrast, the three morphological PCs plotted against δ^15^N values generally revealed a gradient among morphotypes, suggesting some morphological adaptation in relation to trophic level among the four morphs (Figure 5b,d,f). Two‐block partial least‐squares analysis showed a significant relationship between trophic level (δ^15^N) and body shape (*r* = .66; permutation test, *p* ≤ .01) among morphs and a significant first singular axis (SA) (Eigenvalues coefficient = .0060; permutation test, *p* = .03). Head shape was also significantly related to trophic level (δ^15^N) (correlation coefficient = .37; permutation test, *p* ≤ .01) among morphs but had only a marginally significant first singular axis (SA) (Eigenvalues coefficient = .017; permutation test, *p* = .07). Finally, fin and body depth linear measurements and δ^15^N had a significant negative linear relationship (*p* ≤ .01) (Figure 5b).

## 4 Discussion

Linking ecological patterns (e.g., habitat use, feeding tactics) to phenotypic traits (e.g., morphology, behavior) is a fundamental step needed to understand mechanism(s) of origin and maintenance of population differentiation (Martin, Mcgee, & Langerhans, 2015). Our study, applying mixSIAR and nicheROVER models to stable isotope data, explored unresolved questions regarding the potential ecological drivers responsible for the maintenance of Lake Trout polymorphism in Great Bear Lake (Chavarie et al., 2016). Based on our results, selection pressure(s) among the four shallow‐water Lake Trout morphotypes in Great Bear Lake appeared to operate more at the trophic level (i.e., degree of piscivory; δ^15^N values), than at the more commonly reported habitat‐foraging axis (littoral/benthic vs. pelagic; δ^13^C) (Faulks et al., 2015; Parker et al., 2009; Præbel et al., 2013). Our study revealed weak differences in resource use and niche space among the four shallow‐water morphotypes, which raises the question of how polymorphism can be sustained in a species that is known to be an opportunistic generalist feeder (Vander Zanden, Shuter, Lester, & Rasmussen, 2000). The ecological opportunities in Great Bear Lake seem to be linked to the diversity of resources and their availability (e.g., pulsed or limited), coupled with a weak benthic–pelagic gradient in habitat use; these factors favor multiple generalists across a gradient of omnivory.

More specifically, the isotopic evidence suggested a unique combination of multiple generalists versus one specialist (Morph 4) (Amundsen, Gabler, & Staldvik, 1996; Smith, Baumgartner, Suthers, & Taylor, 2011) coexisting within Great Bear Lake. As a general rule, the position and breath of coexisting niches have evolved to match available environmental variation, both spatial and temporal (Kassen, 2002). Niche expansion and flexibility have been commonly observed in lakes with fluctuating resources (e.g., resource turnover) and in species‐poor systems (Bolnick et al., 2010), both of which apply to Great Bear Lake. Indeed, using a broad resource spectrum has been identified as an adaptive strategy for fishes living in Arctic environments, where food availability is patchily distributed and ephemeral (Dill, 1983; Kassen, 2002; Smith et al., 2011). Generalist feeding tactics, illustrated by the larger niche widths of morphs 1–3 than Morph 4, should be suited to taking advantage of Great Bear Lake's low productivity that is concentrated in the littoral zone, typical of many Arctic aquatic ecosystems (Jonsson et al., 1988; MacDonald et al., 2004; Karlsson and Byström; K. Howland, unpublished data). The narrow isotopic niche breadth of Morph 4 confirmed it was the most specialized of the four morphotypes, mainly inhabiting the inshore pelagic environment of Great Bear Lake (Chavarie et al., 2016). Thus, the relative rarity of Morph 4 (Chavarie et al., 2013) might be a reflection of a less favorable feeding strategy in this habitat, given resource productivity and variability over time and space, and may facilitate the presence of multiple generalists (Nonaka, Svanbäck, Thibert‐Plante, Englund, & Brännström, 2015; Svanbäck, Mario Pineda‐Krch, Krch, & Doebeli, 2009; Svanbäck et al., 2015). The observed low abundance of Morph 4 could also reflect a lack of sampling in the profundal zone of Great Bear Lake (>30 m), especially given the limited representation of Morph 4 prey in our sampling. However, preliminary profundal data support the low abundance observed in this study (K. Howland, unpublished data).

Generalist populations (i.e., morphs in this context) may actually consist of subsets of differently specialized individuals, producing a broad population‐level niche as an overall outcome (Bolnick, Yang, Fordyce, Davis, & Svanbäck, 2002; Bolnick et al., 2003; Svanbäck & Bolnick, 2005). Individual specialization in this study may include use of spatially separated resources (i.e., spatial variation) or variable use of resources among years (i.e., temporal variation), both of which could be expected in a depauperate environment such as a large northern lake (Costa et al., 2008; Quevedo, Svanbäck, & Eklöv, 2009; Svanbäck & Persson, 2004). Highly connected trophic networks are known to sustain both opportunistic and selective feeders, for example, sharing preferred resources but differing in alternative resources (Pires et al., 2011). Spatial and temporal variation in ecological opportunities, in addition to individual specialization, seems to occur in Great Bear Lake; however, their extent and their impact on Lake Trout intraspecific diversity remain unknown (Chavarie, Howland, Harris et al., 2015; Chavarie, 2016). The potential for temporal and spatial variation in resource use within a morphotype supports the initial questioning of how intraspecific diversity can be maintained in a generalist forager. Functional traits, for instance within morphology, would be expected to be strongly related to diet due to trade‐offs in foraging efficiency for different prey (Bolnick, Svanbäck, Araújo, & Persson, 2007; Svanbäck & Eklöv, 2002, 2003). However, evidence increasingly suggests that morphology can be a poor proxy for diet specialization, with moderate to weak correlations between the two variables (Bolnick & Paull, 2009; Bolnick et al., 2010).

Overall, our findings contrast with habitat partitioning models associated with horizontal (e.g., littoral–pelagic) or vertical (littoral–profundal) resource axes (McKinnon & Rundle, 2002; Mcphee et al., 2012; Schluter, 1996; Svanbäck & Eklöv, 2002). The lack of clear morphological linkages with trophic adaptations associated with the littoral–pelagic gradient supports the interpretation of widespread use of the shallow‐water zone (≤30 m) by morphs 1–3 in Great Bear Lake. Interestingly, a relationship between morphology and trophic adaptation related to δ^15^N values supports the suggestion that the primary resource axis driving the Lake Trout diversity in Great Bear Lake is linked to the degree of trophic generality versus speciality (see Paull et al. 2012). Even within a shared generalist strategy, variation in niche width and trophic level (δ^15^N) existed among morphs 1–3, suggesting different facets in their generality related to different selective pressures. Those differences could be caused by variation in prey composition and differences in preference for certain prey, as observed from analyses of stomach contents among these Lake Trout morphs (Chavarie et al., 2016). Occurrence of invertebrates versus fish in stomachs varied among morphs 1–3, which seemed to match differences in their isotopic niche widths, for example, Morph 2 had a narrower niche associated with a higher prevalence of fish (cisco). Thus, the ecological opportunity for polymorphism appears not to be associated with habitat partitioning but with the range of prey exploited independent of habitat structure, favoring different degrees of generality among morphs (Martin & Pfennig, 2009; Martin et al., 2015; Pfennig, Rice, & Martin, 2007).

The presence of cannibalism, especially on early life stages, may equalize the benefits of exploiting different resources, ultimately leading to the development of resource polymorphism (Andersson, Bystrom, Claessen, Persson, & De Roos, 2007). Juvenile Lake Trout appear to be cannibalized by all morphs, which could positively influence the maintenance of polymorphisms in this system. Within a population, cannibalism on small‐size classes can indirectly increase the availability of planktonic and benthic resources to larger size classes, expanding their resource base (Andersson et al., 2007; Persson et al., 2004). However, the importance of entering into a piscivorous mode of feeding in early developmental stages, as observed in other systems, may have reduced Lake Trout's ability to handle small prey, thereby decreasing the probability for the development of resource polymorphism relative to other species (Andersson et al., 2007; Collar, O'meara, Wainwright, & Near, 2009; Svanbäck et al., 2015). In addition to cannibalism on juvenile Lake Trout in Great Bear Lake, cannibalism was observed at the egg (C.C. Krueger and A.M. Muir, Pers. Obs.) and adult Lake Trout life stages (Chavarie et al., 2016), which could have a homogenizing effect on isotopic values, potentially reducing detection of resource partitioning. Nonetheless, the apparent prevalence of cannibalism by adult Lake Trout of Great Bear Lake, consistently observed across all dietary methods of measuring trophic interactions (see Chavarie et al., 2016), could indicate that cannibalism is an important driver of the observed generalist–specialist polymorphism. Future research directions should tackle these remaining uncertainties and knowledge gaps.

## 5 Conclusion

Freshwater fishes in high‐latitude environments have provided fruitful systems for understanding mechanisms that promote intraspecific divergence (Mcphee et al., 2012). The ecological theory of adaptive radiation predicts that the evolution of phenotypic diversity within a species will be linked to differential selection arising from using different environments (Kristjansson et al., 2011). In contrast to the different foraging opportunities associated with habitat (e.g., depth) partitioning generally seen in Lake Trout polymorphism across North America (Eshenroder, 2008; Zimmerman et al., 2009), the ecological partitioning in Great Bear Lake seems to operate at the trophic level (δ^15^N), independent of gross differences in habitat. The variability of prey availability over time and space in Arctic lakes, such as Great Bear Lake, appears to favor multiple generalist morphs, with varying degrees of omnivory along a weak benthic–pelagic gradient. This variation in omnivory would account for the overlap found in prey items and the limited niche differentiation among morphs, and explain how this polymorphism can be sustained in an opportunistic generalist feeder. Our study suggested morphological linkages within a gradient of generalization, and thus a form of resource partitioning without large differences in habitat use. Polymorphism in Great Bear Lake seems to depend on several variables, involving functional trade‐offs, resource ephemerality, ecological opportunity, and intensity of intraspecific competition (Pfennig & Pfennig, 2012).

In the context of resource partitioning, Great Bear Lake offers new perspectives in resource polymorphism by demonstrating high intraspecific diversity independent of any clear vertical or horizontal habitat partitioning. In contrast, the rigid divisions of diet specialization among polymorphic Arctic Charr might explain that species’ apparent higher frequency of polymorphism with respect to phenotypic plasticity, type of breeding, behavior, assortative mating, and philopatry than typically observed in Lake Trout (Eshenroder, 2008). Inherent differences in polymorphism between these two congeneric species reflect the complexity of intraspecific diversity patterns and the mechanism(s) by which they occur. We suggest that the multiple generalist morphs of Lake Trout should be considered as a unique form of diversity that challenges the view that multiple specialists is the standard outcome of intraspecific divergence (Kassen, 2002; Abrams, 2006; Svanbäck et al., 2009; Elmer, 2016).

## Conflict of interest

None declared.

## Appendix 1

### 1.1

**Table A1:** Stable isotope values and sample sizes for prey used in mixSIAR analyses; prey taxa ranged from species to family

Prey	*N*	δ^13^C (‰) ± *SD*	δ^15^N (‰) ± *SD*
Agrypnia	2	−21.6 ± 1.6	6.0 ± 0.5
Asynarchus	4	−21.8 ± 3.0	0.7 ± 0.7
Callibaetis	2	−25.2 ± 0.5	2.8 ± 1.1
Chironomidae	14	−20.8 ± 2.9	4.4 ± 1.3
Cisco	11	−26.3 ± 2.7	9.9 ± 0.4
Corixidae	15	−25.3 ± 3.2	4.4 ± 0.9
Cyclops	3	−31.8 ± 0.4	6.1 ± 0.3
Cypriconcha	5	−13.5 ± 4.0	3.7 ± 0.9
Diporeia	17	−24.6 ± 2.7	4.2 ± 0.5
Empididae	3	−22.1 ± 0.7	5.4 ± 1.2
Eurycercus	2	−18.5 ± 6.5	2.0 ± 1.1
Formicidae	8	−27.4 ± 0.2	5.1 ± 1.2
Gammaridae	11	−23.5 ± 1.8	3.3 ± 1.4
Grayling	5	−25.1 ± 1.7	8.7 ± 0.6
Gyraulus	3	−19.5 ± 0.9	4.5 ± 0.3
Heptagenia	3	−26.3 ± 0.4	3.5 ± 0.2
Hyallela	3	−22.1 ± 2.9	3.1 ± 0.6
Kogotus	4	−22.6 ± 1.0	4.3 ± 0.2
Lake Whitefish	18	−22.9 ± 2.8	10.2 ± 0.4
Lepidoptera	1	−28.0	2.4
Leptodiaptomus	72	−31.8 ± 0.8	5.8 ± 0.6
Leptotarsus	15	−21.5 ± 1.6	3.0 ± 0.8
Limnocalanus	19	−33.1 ± 0.7	6.4 ± 0.6
Limnophilus	2	−17.6 ± 3.7	2.5 ± 1.0
Lake Trout Juvenile	13	−22.5 ± 2.1	12.4 ± 0.7
Lymnaea	17	−19.2 ± 4.7	3.6 ± 0.6
Monoporeia	34	−24.9 ± 1.5	3.3 ± 0.8
Mysis	15	−26.3 ± 1.6	6.9 ± 1.1
Nemoura	5	−25.4 ± 1.8	3.5 ± 0.5
Oreodytes	2	−23.1 ± 0.6	4.5 ± 0.8
Orthoptera	3	−26.8 ± 0.2	4.6 ± 0.5
Phrygarea	2	−18.1 ± 1.4	4.3 ± 0.7
Physa	4	−20.5 ± 1.3	4.7 ± 0.3
Pisidium	2	−16.2 ± 0.5	2.8 ± 0.3
Psychoglypha	5	−24.3 ± 2.1	1.6 ± 0.4
Round Whitefish	16	−19.9 ± 1.9	10.1 ± 0.5
Sculpin	13	−21.0 ± 2.1	9.0 ± 1.1
Stickelback	5	−21.8 ± 1.8	6.8 ± 1.0
Stictotarsus	8	−24.0 ± 3.0	5.0 ± 0.7
Tabanus	2	−27.2 ± 0.1	5.8 ± 0.1
Trichoptera	21	−22.4 ± 1.5	6.4 ± 0.7
Valvata	7	−17.1 ± 4.7	4.2 ± 0.6

**Table A2:** Stable isotope values for each morphotype of Lake Trout from Great Bear Lake

	δ^13^C (‰)			δ^15^N(‰)		
	Mean ± *SD*	Min	Max	Mean ± *SD*	Min	Max
Morph 1	−24.1 ± 2.2	−27.6	−18.7	12.1 ± 1.2	10.0	14.4
Morph 2	−24.9 ± 1.7	−27.2	−21.0	13.1 ± 0.7	12.0	14.7
Morph 3	−23.2 ± 2.2	−26.8	−18.2	13.1 ± 0.9	11.3	15.0
Morph 4	−26.5 ± 0.4	−27.3	−25.7	14.1 ± 0.5	13.1	14.8
